# An Antioxidant Extract of the Insectivorous Plant *Drosera burmannii* Vahl. Alleviates Iron-Induced Oxidative Stress and Hepatic Injury in Mice

**DOI:** 10.1371/journal.pone.0128221

**Published:** 2015-05-26

**Authors:** Nikhil Baban Ghate, Dipankar Chaudhuri, Abhishek Das, Sourav Panja, Nripendranath Mandal

**Affiliations:** Division of Molecular Medicine, Bose Institute, P 1/12, Scheme—VIIM, Kolkata, West Bengal, India; University of Calcutta, INDIA

## Abstract

Free iron typically leads to the formation of excess free radicals, and additional iron deposition in the liver contributes to the oxidative pathologic processes of liver disease. Many pharmacological properties of the insectivorous plant *Drosera burmannii* Vahl. have been reported in previous studies; however, there is no evidence of its antioxidant or hepatoprotective potential against iron overload. The antioxidant activity of 70% methanolic extract of *D*. *burmannii* (DBME) was evaluated. DBME showed excellent DPPH, hydroxyl, hypochlorous, superoxide, singlet oxygen, nitric oxide, peroxynitrite radical and hydrogen peroxide scavenging activity. A substantial iron chelation (IC_50_ = 40.90 ± 0.31 μg/ml) and supercoiled DNA protection ([P]_50_ = 50.41 ± 0.55 μg) were observed. DBME also displayed excellent *in vivo* hepatoprotective activity in iron-overloaded Swiss albino mice compared to the standard desirox treatment. Administration of DBME significantly normalized serum enzyme levels and restored liver antioxidant enzymes levels. DBME lowered the raised levels of liver damage parameters, also reflected from the morphological analysis of the liver sections. DBME also reduced liver iron content by 115.90% which is also seen by Perls’ staining. A phytochemical analysis of DBME confirms the presence of various phytoconstituents, including phenols, flavonoids, carbohydrates, tannins, alkaloids and ascorbic acid. Alkaloids, phenols and flavonoids were abundantly found in DBME. An HPLC analysis of DBME revealed the presence of purpurin, catechin, tannic acid, reserpine, methyl gallate and rutin. Purpurin, tannic acid, methyl gallate and rutin displayed excellent iron chelation but exhibited cytotoxicity toward normal (WI-38) cells; while DBME found to be non-toxic to the normal cells. These findings suggest that the constituents present in DBME contributed to its iron chelation activity. Additional studies are needed to determine if DBME can be used as a treatment for iron overload diseases.

## Introduction

Free radicals, such as reactive oxygen species (ROS) and reactive nitrogen species (RNS), play a significant role in the early onset of oxidative stress and are capable of damaging biologically relevant molecules, such as proteins, nucleic acids and plasma membrane lipids [1]. Antioxidants can interrupt the chain reaction cycle (oxidation process) via different mechanisms, such as chelating metals that catalyze the formation of free radicals and scavenging the free radicals. Therefore, antioxidants are vital for the human body due to their ability to combat oxidative damage [2]. Iron is a metal that is needed by all mammalian cells for growth and survival [3]; however, its extreme deposition can increase oxidative stress in the liver and lead to further injuries, such as hepatocellular necrosis [4], inflammation [5], fibrosis [6,7] and cancer [8]. The human body is largely dependent on the liver for the facilitation of many vital biochemical pathways that manage growth, nutrient supply, energy provision, reproduction and defense [9]. Liver damage (hepatotoxicity) caused by iron overload hinders these processes and can result in serious health problems [10]. Iron removal by chelation therapy is an effective life-saving strategy for nearly all the aforementioned iron overload-induced diseases. Several synthetic iron chelating agents, such as deferoxamine, 1,2-dimethyl-3-hydroxypyrid-4-one (deferiprone, L1) and deferasirox, are available for clinical use; however, these drugs possess several undesirable side effects [11,12]. Thus, the scientific community continues to search for a raw material or isolated natural product that can act as an antioxidant and iron chelator without adverse effects.

*Drosera burmannii* Vahl. (family Droseraceae) is an acaulescent insectivorous herb commonly known as sundew that belongs to one of the largest genera of carnivorous plants, with over 105 species. This herb is distributed throughout the Indian subcontinent as well as China, Australia and West Africa, and it is reported to possess rubefacient properties [13]. Moreover, antifertility [14], anticonvulsant [15] and antitumor activities in mice [16] were reported in the alcohol and aqueous extracts of *D*. *burmannii*. Different species of Drosera contain several medicinally active compounds, including 1,4-naphthoquinones such as plumbagin [17], hydroplumbagin glucoside [18], flavonoids (kaempferol, myricetin, quercetin and hyperoside) [19] and rossoliside (7-methyl-hydrojuglone-4-glucoside). Previous literature reports that several species of Drosera are used in various traditional and homeopathic treatments. Some of the species of this family are used for the treatment of cough in Asia. This plant was also listed in *French Pharmacopoeia* in 1965 for the treatment of chronic bronchitis, asthma and Whooping cough [20]. The present study aimed to assess the *in vitro* antioxidant and *in vivo* hepatoprotective properties against iron-overload-induced liver toxicity in Swiss albino mice.

## Materials and Methods

### Chemicals

2,2′-azinobis-(3-ethylbenzothiazoline-6-sulfonic acid) (ABTS) was procured from Roche diagnostics, Mannheim, Germany. 6-hydroxy-2,5,7,8-tetramethylchroman-2-carboxylic acid (Trolox) was obtained from Fluka, Buchs, Switzerland. Potassium persulfate (K_2_S_2_O_8_), 2-deoxy-2-ribose, ethylene diammine tetraacetic acid (EDTA), ascorbic acid, trichloroacetic acid (TCA), mannitol, nitro blue tetrazolium (NBT), reduced nicotinamide adenine dinucleotide (NADH), phenazine methosulfate (PMS), sodium nitroprusside (SNP), 1,10-phenanthroline, sulphanilamide, N-(1-Naphthyl)ethylenediamine dihydrochloride (NED), L-histidine, lipoic acid, sodium pyruvate, quercetin, ferrozine glutathione reduced, bathophenanthrolinesulfonate disodium salt and 5,5′-dithiobis-2-nitrobenzoic acid (DTNB) were obtained from Sisco Research Laboratories Pvt. Ltd, Mumbai, India. HPLC grade acetonitrile, ammonium acetate, hydrogen peroxide, potassium hexacyanoferrate, Folin-ciocalteu reagent, sodium carbonate, mercuric chloride, potassium iodide, anthrone, vanillin, thiourea, 2,4-dinitrophenylhydrazine (DNPH), sodium hypochlorite, aluminum chloride, xylenol orange, butylated hydroxyltoluene (BHT), N,N- dimethyl-4-nitrosoaniline ammonium iron (II) sulfatehexahydrate [(NH_4_)2Fe(SO_4_)_26_H_2_O], 1-chloro-2,4-dinitrobenzene (CDNB), chloramine-T, hydroxylamine hydrochloride and Dimethyl-4-aminobenzaldehyde were procured from Merck, Mumbai, India. 2,2-diphenyl-1-picrylhydrazyl (DPPH), ferritin, methyl gallate, tannic acid, rutin, gallic acid, (+) catechin and curcumin were obtained from MP Biomedicals, France. Catalase, reserpine, streptomycin sulfate and sodium bicarbonate were obtained from HiMedia Laboratories Pvt. Ltd, Mumbai, India. Evans blue was purchased from BDH, England. D-glucose was procured from Qualigens Fine Chemicals, Mumbai. Diethylenetriaminepentaacetic acid (DTPA) was obtained from Spectrochem Pvt. Ltd, Mumbai, India. Thiobarbituric acid (TBA) was obtained from Loba Chemie, Mumbai, India. Iron-dextran and guanidine hydrochloride was purchased from Sigma-Aldrich, USA. The standard oral iron chelating drug, desirox, was obtained from Cipla Ltd., Kolkata, India.

### Ethics

A sample of the insectivorous plant *Drosera burmannii* Vahl. was collected in January 2014 from public areas adjoining villages in the *Bankura* district in the state of West Bengal, India. These areas are not within a National Park/Reserve Forest/Govt. protected area, and only verbal permission from village headmen was obtained before collection. The conservation status of this insectivorous plant was classified using the International Union for Conservation of Nature (IUCN) World Conservation Union guidelines (1994). The status was ‘LC; Least concern’ as per IUCN red list criteria. The material collected for this study was sampled on a very limited scale and therefore had negligible effects on broader ecosystem functioning.

All animal experiments (Swiss albino mice) were performed in strict accordance with the recommendations of the Committee for the Purpose of Control and Supervision of Experiments on Animals (CPCSEA), Ministry of Environment and Forest, Govt. of India (Bose Institute Registration. No. 95/1999/CPCSEA). The protocol was approved by the Institutional Animal Ethics Committee, Bose Institute. All surgery was performed under ethyl ether anesthesia, and all efforts were made to minimize suffering.

### Animals

Male Swiss albino mice (20 ± 2 g) were purchased from Chittaranjan National Cancer Institute (CNCI), Kolkata, India and were maintained under a constant 12-h dark/light cycle at an environmental temperature of 22 ± 2°C. Mice were provided a normal laboratory pellet diet and water *ad libitum*. The condition of the animals were monitored every 6-h after the treatment and there were no unintended animal deaths during the experimental procedures.

### Plant extract preparation

*D*. *burmannii* was authenticated by the Botanical Survey of India, Kolkata, India. Samples were sorted, cleaned of substratum and shadow dried for extraction. The dried sample (100 g) was then powdered and stirred using a magnetic stirrer with 70% methanol in water (1000 ml) for 15 hours. The mixture was then centrifuged at 2,850 *g*, and the supernatant was decanted. The process was repeated by adding more solvent to the precipitated pellet. The supernatants from the two phases were mixed, concentrated in a rotary evaporator at 40°C, lyophilized and labeled as DBME. The dried extract was stored at -20°C until use.

### In vitro study

#### Total antioxidant activity and reducing power

The total antioxidant capacity of DBME was evaluated by an ABTS•^+^ radical cation decolorization assay in comparison to a trolox standard [21] and 2,2-diphenyl-1-picrylhydrazyl (DPPH) radical scavenging assay [22]. The reducing power of DBME was determined using a previously described method [21].

#### Reactive oxygen species (ROS) scavenging activity

The ROS scavenging ability of DBME was determined using multiple stable ROS radical scavenging assays, such as hydroxyl, superoxide, hypochlorous radical, singlet oxygen and hydrogen peroxide assays by standard procedures [21].

#### Reactive nitrogen species (RNS) scavenging activity

The RNS scavenging activity of DBME was determined using nitric oxide and peroxynitrite radical scavenging assays [21].

#### Iron chelation assay

The Fe^2+^ chelating activity of DBME, catechin, methyl gallate, purpurin, reserpine, rutin and tannic acid was evaluated according to standard methods [23]. HEPES buffer (20 mM, pH 7.2), DBME (0–120 μg/ml) and a positive control EDTA (0–20 μg/ml) were separately added to a 12.5 μM ferrous sulfate solution, and 75 μM ferrozine was added to start the reaction. The mixture was shaken vigorously and left standing for 20 min at room temperature. Next, the absorbance was measured at 562 nm. All tests were performed six times.

#### DNA protection assay

DNA protection was studied using supercoiled pUC18 plasmid DNA according to previously described methods [24] with minor modifications. In HEPES buffer, (pH 7.2, 13 mM), a FeSO_4_ solution (15 μM), DBME of varying doses (0–100 μg), DNA (1 μg) and water were added to produce an initial reaction mixture. Next, an H_2_O_2_ solution (0.0125 mM) was added to start the reaction. After 10 min, the reaction was stopped by adding desferal (0.2 mM) followed by a loading buffer. Each reaction mixture (20 μl) was loaded in a 1% agarose gel. After migration, the gel was stained with ethidium bromide and visualized with a UV transilluminator. The DNA bands were quantified using densitometry, and the following formulas were used to calculate the protection percentage:

%SC=[1.4X SC/(OC+(1.4X SC))]X100and

%protection=100X[(control SC−chelator SC)/(control SC−no chelator SC)−1],

where SC = supercoiled; OC = open circular; 1.4 = correction factor.

The ability of the plant extract to protect DNA supercoils can be expressed as the [P]_50_ value, which is defined by the concentration of sample required for 50% protection.

#### Ferritin iron release assay

This assay was performed according to a previously described method [25]. The release of ferritin iron was measured using the ferrous chelator ferrozine as a chromophore. The reaction mixture contained 200 μg ferritin and 500 μM ferrozine in 50 mM phosphate buffer with a pH of 7.0. The reaction was started by the addition of DBME at different concentrations (100–500 μg), and the change in absorbance was measured continuously at 560 nm for 20 min. A cuvette containing ferritin, ferrozine and phosphate buffer but no plant extract was used as the reference solution.

### In vivo study

#### Experimental design

Mice were divided into six groups of six mice each. One group was labeled as blank (B) and received normal saline. The other five groups received five intraperitoneal injections of iron-dextran at a dose of 100 mg/kg b.w. (one dose every two days). One iron-dextran group (C) was administered normal saline, and the other four groups were orally treated with either 50 mg/kg b.w. (S50), 100 mg/kg b.w. (S100), 200 mg/kg b.w. (S200) DBME or 20 mg/kg b.w. desirox (D) for 21 days beginning on the day following the first iron-dextran injection.

#### Sample collection and tissue preparation

After treatment, mice were fasted overnight on the 21^st^ day and anesthetized with ethyl ether. Blood was collected by cardiac puncture. Serum from the blood samples was separated using a cooling centrifuge and stored at -80°C until analysis. The liver was washed with ice-cold saline and divided into three parts. One major portion of the liver was cut, weighed and homogenized in 10 volumes of 0.1 M phosphate buffer (pH 7.4) containing 5 mM EDTA and 0.15 M NaCl. The sample was centrifuged at 8,000 *g* for 30 min at 4°C. The protein concentration in the supernatant was estimated according to Lowry's method using BSA as a standard. The remaining samples were stored at -80°C until further analysis. Another portion of the liver was weighed and digested using an equivolume mixture of sulfuric acid and nitric acid for iron content analysis. A final liver sample was used for histopathological studies.

#### Serum markers and ferritin levels

Alanine amino transferase (ALAT), aspartate amino transferase (ASAT), and bilirubin levels were measured in serum samples using commercial kits from Merck, Mumbai, India. Serum alkaline phosphatase (ALP) was estimated using a kit supplied by Sentinel diagnostics, Italy. The serum ferritin level was measured using an enzyme-linked immunosorbent assay (Monobind Inc., USA) according to the manufacturer’s instructions.

#### Antioxidant enzymes

Superoxide dismutase (SOD) [26], catalase (CAT) activity [27], glutathione-S-transferase (GST) [28] and reduced glutathione (GSH) levels [29] were assayed according to previously reported methods.

Lipid peroxidation products, protein carbonyl content, hydroxyproline and liver iron content

The lipid peroxide levels [30], protein carbonyl content, hydroxyproline content [31] and liver iron levels [32] were measured in samples according to standardized methods.

#### Histopathological analysis

The liver samples were excised, washed with normal saline, and processed separately for histological study. Initially, the material was fixed in 10% buffered neutral formalin for 48 h. The samples were then paraffin-embedded, and sections with a 5-μm thicknesses were stained with hematoxylin and eosin (morphological examination), Perls’ Prussian blue dye (iron content) and Masson’s trichrome stain (liver fibrosis). The stained sections were examined for histopathological changes under a light microscope.

### Phytochemical and high performance liquid chromatography (HPLC) analyses of DBME

The analysis of resident phytochemicals, including alkaloids, carbohydrates, flavonoids, glycosides, phenols, saponins, tannins, terpenoids, anthraquinones and triterpenoids, in the extract was completed using standard qualitative and quantitative methods as previously described [33,34]. For HPLC analysis, standard stock solutions (10 μg/ml) were prepared in mobile phase for PRME, purpurin, catechin, tannic acid, reserpine, methyl gallate and rutin. All the samples were filtered through a 0.45-μm polytetrafluoroethylene (PTFE) filter (Millipore) to remove any particulate matter. The analysis was performed using a HPLC-Prominence System RF10AXL (Shimadzu Corp.) equipped with a degasser (DGU-20A5), quaternary pump (LC-20AT), auto-sampler (SIL-20A) and detectors of reflective index (RID-10A), fluorescence (RF-10AXL) and diode array (SPD-M20A). A 20 μl aliquot of each sample and standard was injected and analyzed in triplicate. Gradient elution consecutive mobile phases of acetonitrile and 0.5 mM ammonium acetate in water at a flow rate of 1 ml/min for 65 min through the column (ZIC-HILIC) was maintained at 25°C. The detection was completed at 254 nm.

### WST-1 cytotoxicity assay

The human lung fibroblast (WI-38) cell line was purchased from the National Centre for Cell Science (NCCS), India. Cells were grown in DMEM supplemented with 10% (v/v) fetal bovine serum (FBS), 100 U/ml penicillin G, 50 μg/ml gentamycin sulfate, 100 μg/ml streptomycin and 2.5 μg/ml amphotericin B. The cell line was maintained in a CO_2_ incubator at 37°C in a humidified atmosphere containing 5% CO_2_. Cell proliferation and cell viability were quantified using the WST-1 Cell Proliferation Reagent, Roche diagnostics, according to previously described methods [35]. For this experiment with pure compounds (purpurin, catechin, tannic acid, reserpine, methyl gallate and rutin), a 2 mg/ml aqueous solution with 0.2% DMSO is used; in such a way that DMSO concentration in the cell culture media did not exceed 4 x 10^–4^%, thus being non-toxic to the cells. Briefly, WI-38 cells (1 × 10^4^ cells/well) were treated with DBME, purpurin, catechin, tannic acid, reserpine, methyl gallate or rutin at doses ranging from 0–120 μg/ml for 48 hours in 96-well culture plates. After treatment, 10 μl of the WST-1 cell proliferation reagent was added to each well followed by 2 hours of incubation at 37°C. Cell proliferation and viability were quantified by measuring the absorbance at 460 nm using a microplate ELISA reader MULTISKAN EX (Thermo Electron Corporation, USA).

### Statistical analysis

All data are reported as the mean ± SD of six measurements. The statistical analysis was performed using KyPlot version 2.0 beta 15 (32 bit) and Origin professional 6.0. Comparisons among groups were assessed with paired *t*-tests. In all analyses, a *p* value of <0.05 was considered significant.

## Results and Discussion

### In vitro study

#### DBME acts as a potent antioxidant and free radical scavenger

**Fig 1:**
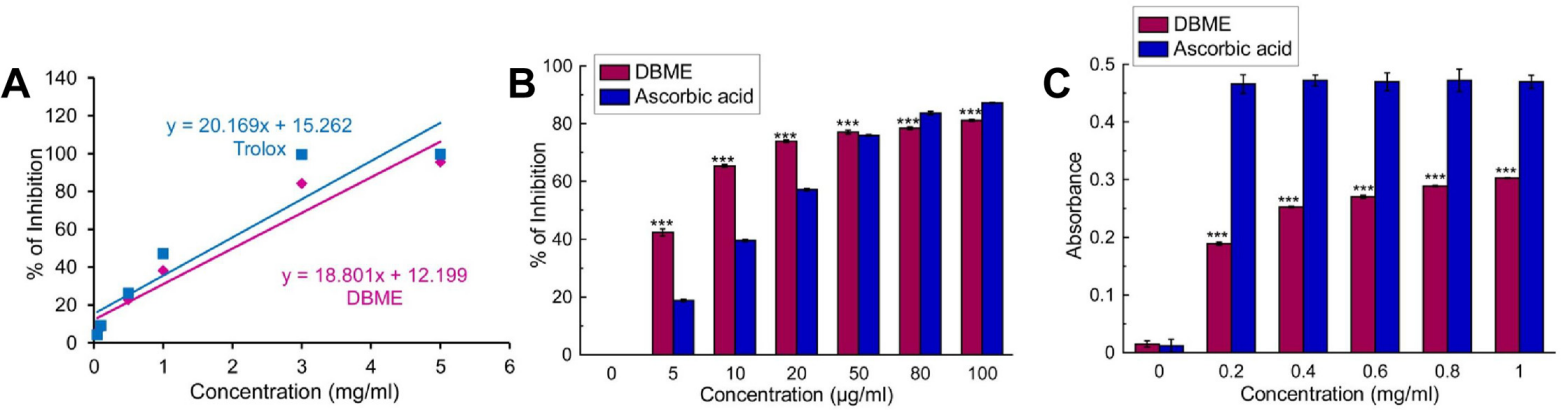
Total antioxidant activity and reducing power of DBME and the reference compounds. (A) total antioxidant activity, (B) DPPH radical scavenging, (C) reducing power activity. The results are mean ± S.D. of six parallel measurements. ***p < 0.001 vs. 0 μg/ml.

**Fig 2:**
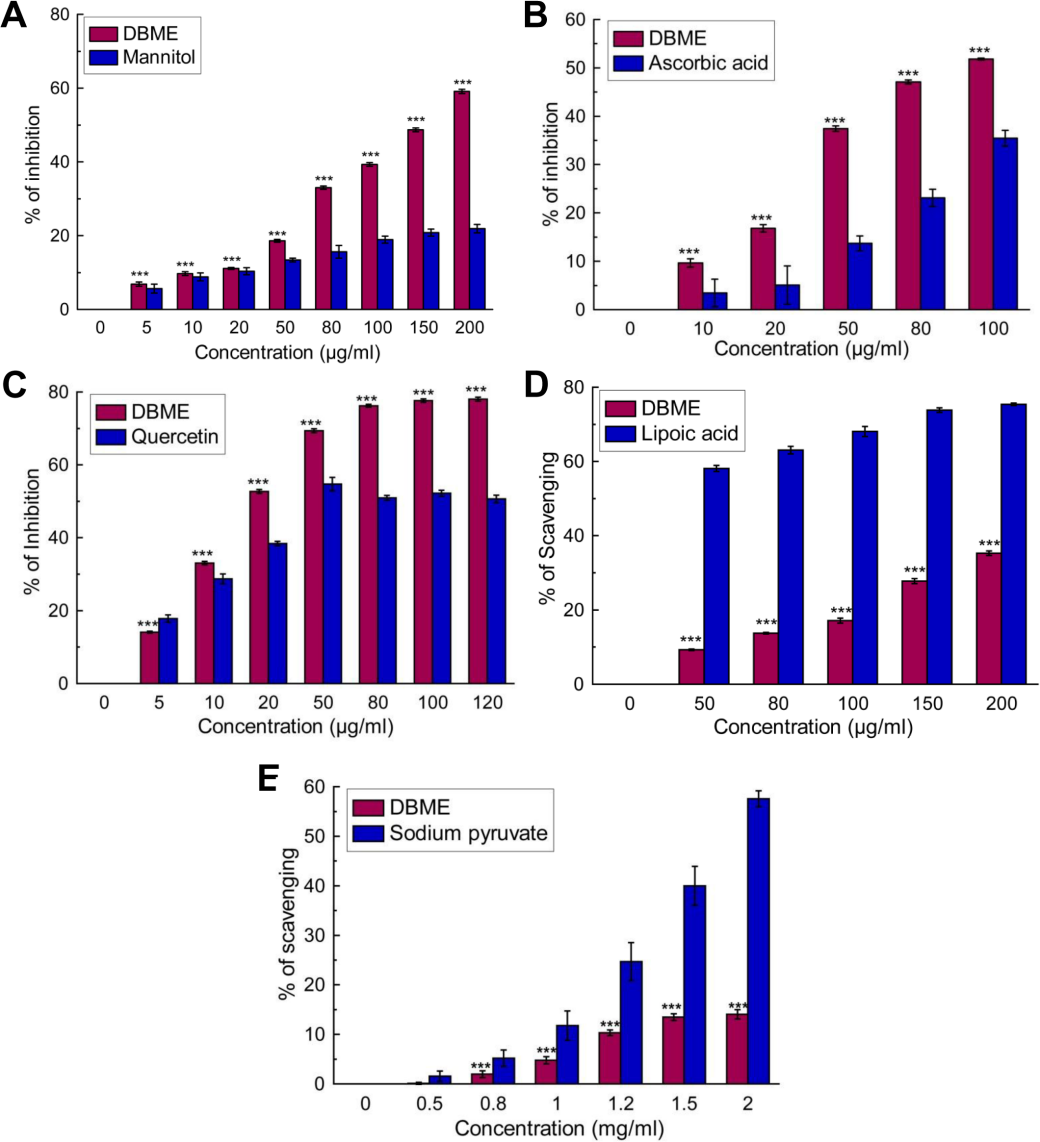
Reactive oxygen species scavenging activity of DBME and the reference compounds. (A) Hydroxyl radical inhibition, (B) hypochlorous radical scavenging, (C) superoxide radical inhibition, (D) singlet oxygen radical scavenging, (E) hydrogen peroxide scavenging. The results are mean ± S.D. of six parallel measurements. ***p < 0.001 vs. 0 μg/ml.

**Fig 3:**
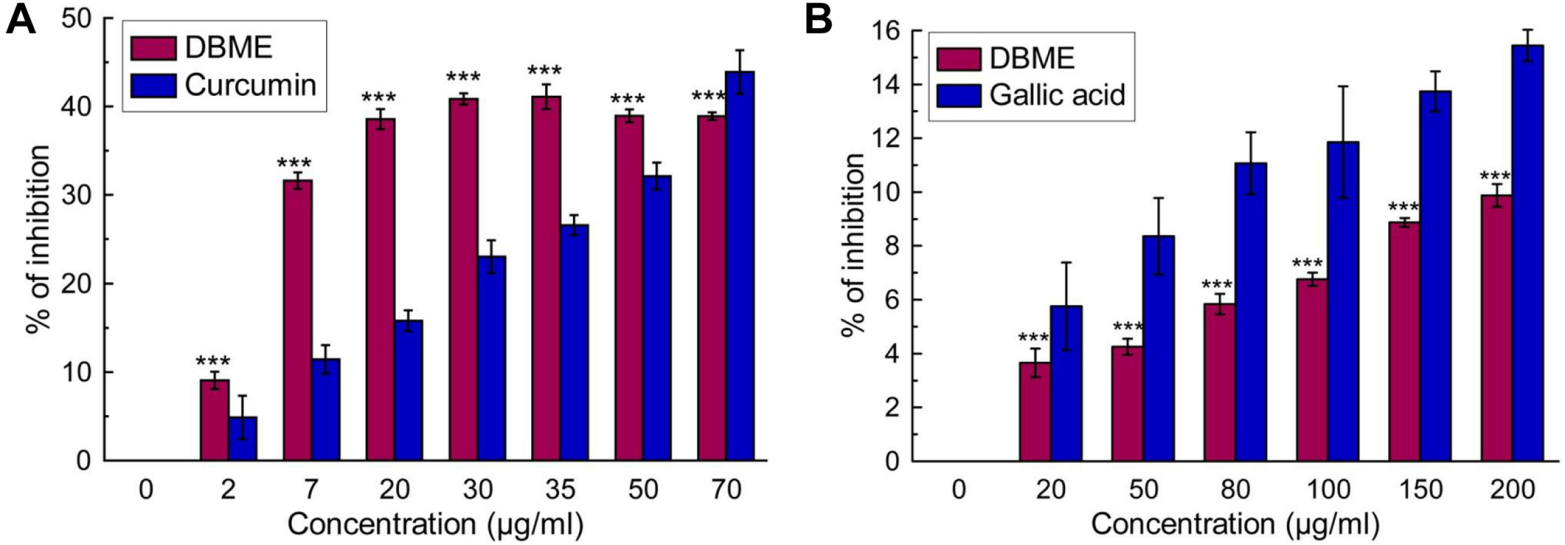
Reactive nitrogen species scavenging activity of DBME and the reference compounds. (A) Nitric oxide inhibition, (B) peroxynitrite radical scavenging. The results are mean ± S.D. of six parallel measurements. ***p < 0.001 vs. 0 μg/ml.

The overall total antioxidant activity of DBME was measured, and the trolox (standard) equivalent antioxidant capacity (TEAC) was 0.932 ± 0.008 (Fig 1A). This result demonstrates that the extract possesses significant antioxidant properties. DBME was then investigated for DPPH radical, a stable free radical and accepted widely as a system for estimating antioxidative capacity, scavenging as well as reducing power capacity. We found that DBME possesses an excellent dose-dependent scavenging activity for the DPPH radical (Fig 1B) and showed a promising reducing power (Fig 1C). The observed DPPH radical scavenging activity was similar to that of the ascorbic acid standard. Several ROS are generated in our body systems, including hydroxyl, hypochlorous, superoxide, singlet oxygen and hydrogen peroxide radicals. The hydroxyl radical causes enormous damage to the biomolecules of living cells [36]. Similarly, hypochlorous acid is produced at the sites of inflammation due to the oxidation of Cl¯ ions by the neutrophil enzyme myeloperoxidase [37] and induces target cell lysis, inactivates [alpha1]-antiprotease, activates collagenase and gelatinase, depletes antioxidant vitamins, such as ascorbic acid, and inactivates antioxidants enzymes, such as catalase [38]. Similarly, the superoxide anion is considered to be a harmful reactive oxygen species. DBME scavenged these three ROS (Fig 2A, 2B and 2C) with IC_50_ values lower than the respective standards. Singlet oxygen, a high energy form of oxygen, is generated in the skin upon UV-radiation and induces hyperoxidation and oxygen cytotoxicity and decreases the antioxidant activity [39]. Hydrogen peroxide itself is not very reactive but can cause cytotoxicity by producing hydroxyl radicals in the cell [40]. Fig 2D demonstrates that the singlet oxygen radical scavenging activity of DBME is approximately equal to the standard; however, DBME is less effective at scavenging hydrogen peroxide (Fig 2E). Nitric oxide can mediate toxic effects through DNA fragmentation and cell damage and plays a role in multiple sclerosis, arthritis, juvenile diabetes, neuronal cell death, inflammatory conditions and different carcinomas when overproduced [41]. Moreover, the toxic effect of NO greatly increases after reacting with the superoxide radical to form the highly reactive peroxynitrite anion (ONOO¯). In the present study, DBME showed significant scavenging activity against both nitric oxide (Fig 3A) and peroxynitrite radicals (Fig 3B). The IC_50_ values for DBME on ROS and RNS scavenging are shown in Table 1 with the respective standard compounds.

**Table 1:** IC_50_ values of the DBME and standard compounds for DPPH, ROS and RNS scavenging and iron chelation assays.

Name of Assay	DBME	Standard	Values of Standard compounds
DPPH	7.56 ± 0.23	Ascorbic acid	5.29 ± 0.28
Hydroxyl radical (OH^•^) scavenging	157.81 ± 1.24	Mannitol	571.45 ± 20.12
Superoxide anion (O_2_^•−^) scavenging	22.86 ± 0.45	Quercetin	42.06 ± 1.35
Hypochlorous acid (HOCl) scavenging	90.17 ± 1.64	Ascorbic acid	235.96 ± 5.75
Singlet oxygen (^1^O_2_) scavenging	414.40 ± 5.88	Lipoic acid	46.16 ± 1.16
Hydrogen peroxide (H_2_O_2_) scavenging	12.93 ± 0.63	Sodium pyruvate	3.24 ± 0.30
Nitric oxide radical (NO) scavenging	55.09 ± 1.31	Curcumin	90.82 ± 4.75
Peroxynitrite (ONOO^-^) scavenging	1.55 ± 0.48	Gallic acid	0.876 ± 0.57
Iron chelating activity	40.90 ± 0.31	EDTA	1.27 ± 0.05

# IC_50_ values of all activities are determined in μg/ml except in hydrogen peroxide and peroxynitrite scavenging assays where values express in mg/ml. Data expressed as mean ± S.D (n = 6). EDTA represents Ethylenediamine tetraacetic acid.

#### DBME acts as an effective iron chelator and DNA protector

**Fig 4:**
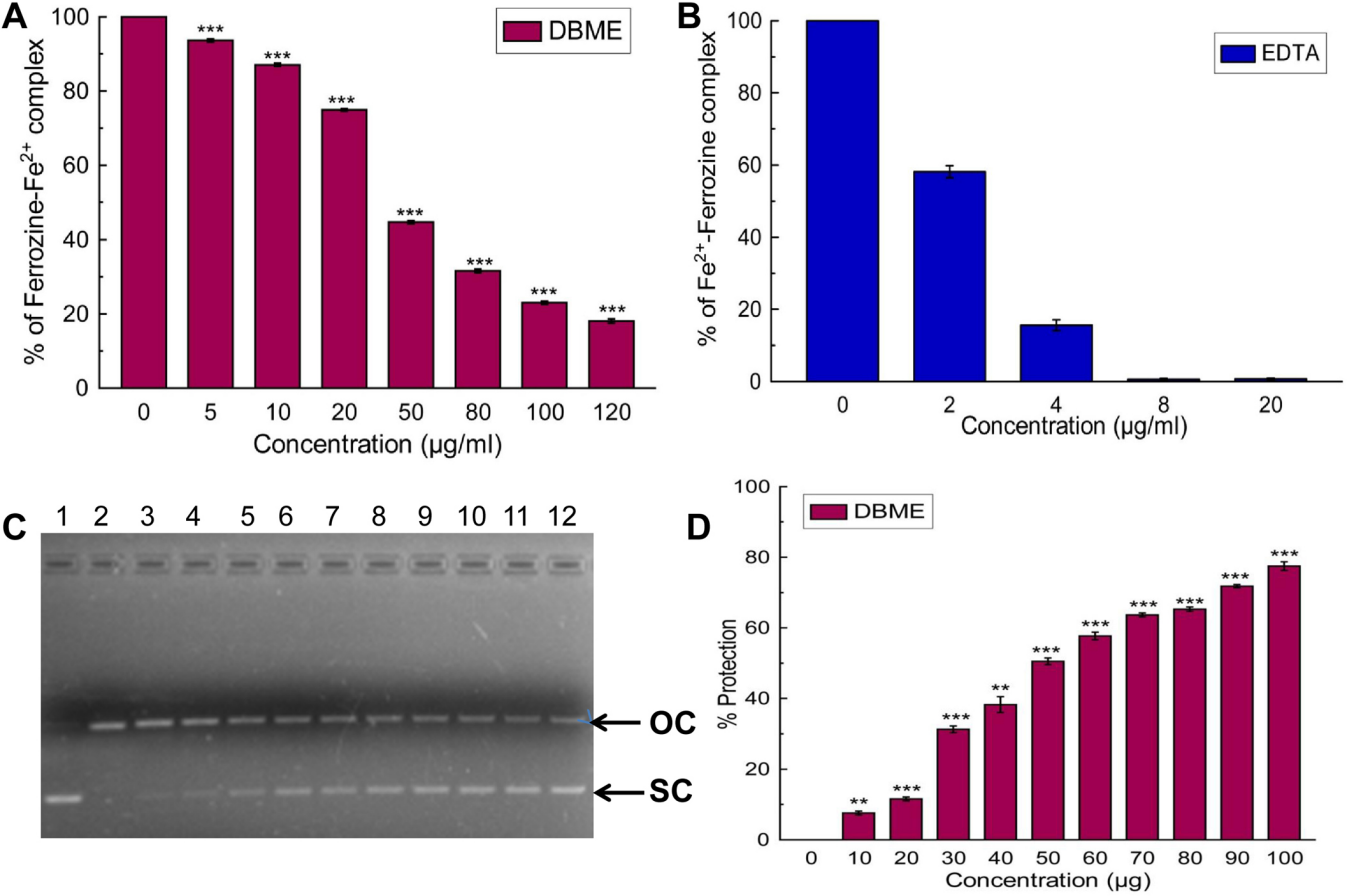
Iron chelation activity of DBME. (A) *In vitro* iron chelation acitivity of DBME. (B) *In vitro* iron chelation activity of EDTA. (C) Picture of agarose gel of pUC-18 DNA showing bands of supercoiled (SC) and open circular (OC) forms. Lanes on the gel represent: (Lane 1) control DNA (no H_2_O_2_ or Fe^2+^); (Lane 2) reaction mixture without extract; (Lane 3–12) reaction mixtures with extract of increasing concentrations (10–100 μg) (D) Graphical representation of % of supercoiled DNA protection by DBME. The results are mean ± S.D. of six parallel measurements. **p < 0.01 and ***p < 0.001 vs 0 μg/ml.

The ability to chelate Fe^2+^ was determined by disruption of the formation of violet colored Fe^2+^-ferrozine by DBME and the reference compound EDTA (Fig 4A and 4B). The IC_50_ values of DBME and EDTA were calculated to be 40.90 ± 0.30 and 1.27 ± 0.05 μg/ml, respectively. The highest dose of DBME inhibited complex formation up to 82%. Moreover, free iron takes part in the initiation and propagation of various ROS, which eventually results in oxidative damage to several vital biomolecules, the peroxidation of membrane lipids, mitochondrial damage, DNA fragmentation and, ultimately, cell death [42]. DBME dose-dependently protected against the scission of pUC 18 plasmid DNA (Fig 4C). pUC 18 supercoiled DNA was used as a control (lane 1). Lane 2 was composed of the open circular form of DNA generated by Fenton's reaction. Addition of DBME resulted in the restoration of DNA in the supercoiled form (lane 3–12). The results in Fig 4D show the dose-dependent DNA protection by DBME with a [P]_50_ value of 50.41 ± 0.55 μg. Previous observations suggested that extracts with the ability to chelate free iron and protect against Fenton reaction-mediated supercoiled DNA damage *in vitro* would show promising *in vivo* iron chelation and hepatoprotective activity [31,43,44]. Our current *in vitro* results suggest that DBME is an excellent antioxidant, especially for hydroxyl radical scavenging; furthermore, DBME shows a strong iron chelating ability.

### In vivo study

#### DBME normalized the serum markers that were increased after hepatocellular injury

As shown in Table 2, severe acute hepatic damage (hemochromatosis) produced by the intraperitoneal administration of iron-dextran leads to the leakage of the cellular enzymes into the bloodstream and a significant elevation in the levels of serum ALAT, ASAT, ALP, and bilirubin in mice. Hepatic damage from iron overload leads to increased levels of serum ALAT, ASAT, ALP, and bilirubin [3]. After treatment with DBME, the levels of these enzymes and bilirubin were significantly lowered even when compared with the standard iron chelator drug desirox. The results suggest that treatment with DBME ameliorated the iron overload-induced hepatic damage in mice.

**Table 2:** The effect of DBME on serum markers (ALAT, ASAT, ALP, bilirubin) in iron overloaded mice.

Treatment	ALAT (Unit/l)	% Change	ASAT (Unit/l)	% Change	ALP (Unit/l)	% Change	Bilirubin (mg/dl)	% Change
**B**	15.25 ± 1.56	-	67.60 ± 5.78	-	33.08 ± 3.71	-	1.52 ± 0.09	-
**C**	45.23 ± 2.45^c^	196.63	167.62 ± 4.64^c^	147.91	132.51 ± 6.42^c^	300.57	3.28 ± 0.20^c^	116.29
**S50**	38.38 ± 0.42^c^ ^e^	151.72	105.44 ± 2.98^c^ ^f^	55.95	107.88 ± 3.45^c^ ^f^	226.11	3.03 ± 0.65^b^	100.23
**S100**	28.43 ± 0.29^c^ ^f^	86.45	99.04 ± 3.30^c^ ^f^	46.48	64.89 ± 4.88^c^ ^f^	96.17	2.13 ± 0.24^b^ ^f^	40.27
**S200**	20.77 ± 0.48^c^ ^f^	36.19	81.09 ± 3.09^b^ ^f^	19.94	38.66 ± 3.72^b^ ^f^	16.86	1.77 ± 0.13^b^ ^f^	16.40
**D**	23.29 ± 1.09^c^ ^f^	52.79	79.35 ± 4.36^a^ ^f^	17.36	61.01 ± 2.44^c^ ^f^	84.44	1.68 ± 0.15^f^	10.97

Values are mean ± SD of six observations.

^a^ *p<*0.05

^b^ *p<*0.01 and ^c^ *p<*0.001 significant difference from normal mice (B) group

^e^ *p<*0.01 and ^f^ *p*<0.001significant difference from iron overloaded (C) group

#### DBME enhanced the levels of liver antioxidants

The activity of antioxidant enzymes SOD, CAT and GST and non-enzymatic antioxidant was significantly reduced in iron overloaded mice. These antioxidants are the body’s intrinsic defense mechanism against oxidative stress [45]. After orally treating the iron overloaded mice with DBME, the levels of antioxidants were significantly increased, and the highest dose of DBME showed superior effects compared with desirox (Table 3).

**Table 3:** The effect of DBME on liver parameters (SOD, CAT, GST, GSH) in iron overloaded mice.

Treatment	SOD (Unit/mg protein)	% Change	CAT (Unit/mg protein)	% Change	GST (Unit/mg protein)	% Change	GSH (μg/mg protein	% Change
**B**	0.55 ± 0.03	-	21.84 ± 3.12	-	5.76 ± 0.29	-	0.51 ± 0.02	-
**C**	0.09 ± 0.04^c^	84.09	7.74 ± 2.5^c^	64.56	1.50 ± 0.3^c^	73.96	0.36 ± 0.01^c^ ^3^	28.40
**S50**	0.10 ± 0.05^c^	81.62	14.12 ± 2.73^c^ ^f^	35.35	2.50 ± 0.26^c^ ^d^	56.59	0.37 ± 0.01^c^	26.63
**S100**	0.29 ± 0.05^c^ ^f^	48.29	16.16 ± 2.68^c^ ^f^	26.01	3.25 ± 0.3^c^ ^e^	43.58	0.43 ± 0.03^b^ ^e^	14.79
**S200**	0.52 ± 0.05^f^	6.31	18.36 ± 2.29^c^ ^f^	15.93	3.86 ± 0.32^c^ ^f^	32.99	0.46 ± 0.02^c^ ^f^	8.87
**D**	0.47 ± 0.05^a^ ^f^	15.31	16.14 ± 3.87^c^ ^f^	26.09	3.72 ± 0.36^c^ ^f^	35.42	0.48 ± 0.02^a^ ^f^	5.13

Values are mean ± SD of six observations.

^a^ *p<*0.05, ^b^ *p<*0.01and ^c^ *p<*0.001 significant difference from normal mice (B) group

^d^ *p<*0.05, ^e^ *p<*0.01 and ^f^ *p<*0.001 significant difference from iron overloaded (C) group

#### DBME normalised an increase in biochemical parameters

**Fig 5:**
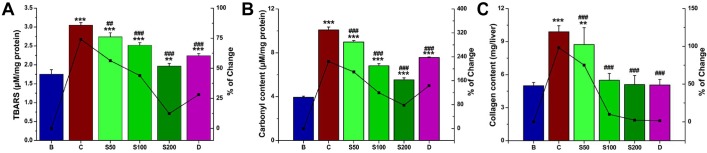
Effect of DBME on biochemical parameters. (A) Hepatic lipid peroxidation levels, (B) protein oxidation levels, (C) collagen content. Mice were randomly divided into six groups (blank, B; control, C; 50 mg/kg b.w. DBME, S50; 100 mg/kg b.w. DBME, S100; 200 mg/kg b.w. DBME, S200; desirox group, D) and treated as described in ‘experimental design’ section. Values are expressed as mean ± SD of six mice. **p < 0.01, ***p < 0.001 compared with blank and ##p < 0.01, ###p < 0.001 compared with control.

Iron induced pathogenicity in the liver leads to structural and functional alterations in cell integrity by enhancing the lipid peroxidation. Thiobarbituric acid reactive substances (TBARS) is the end product of lipid peroxidation, and an elevated TBARS level is used as a main marker of hepatocellular injury [46]. The TBARS level was increased (74%) as a result of the oxidative stress in iron overloaded mice compared to the normal mice. After treatment with increasing doses of DBME, TBARS substantially decreased to a normal level (Fig 5A). Another consequence of liver damage is the excessive accumulation of extracellular proteins, such as collagen, and a significant increase in the amount of hydroxyproline due to liver fibrosis [47]. Fig 5B and 5C reflect the elevation in protein carbonyl (155%) and hydroxyproline (138%) contents, respectively, in iron overloaded mice compared to normal mice. When treated with DBME, these two liver damage markers significantly and dose-dependently normalized. Overall, DBME is more effective at ameliorating the liver injury/ fibrosis caused by iron overload than the standard iron chelating drug desirox.

#### DBME decreased the levels of liver iron and serum ferritin

**Fig 6:**
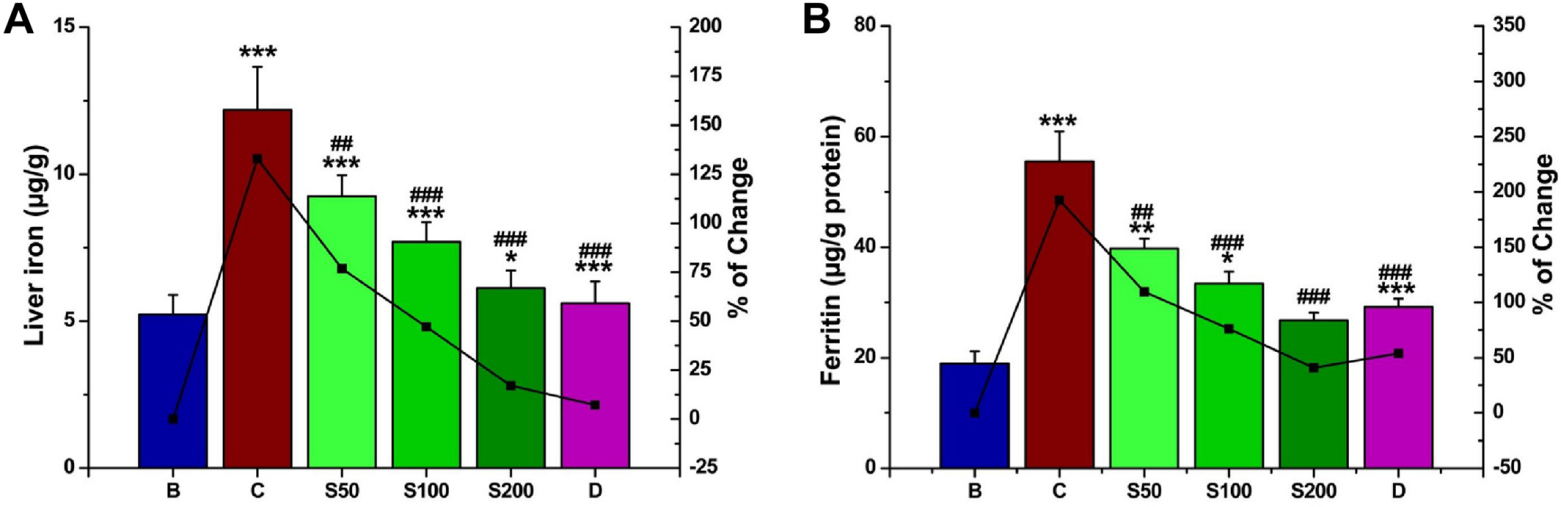
Effect of DBME on hepatic iron content and serum ferritin level. (A) hepatic iron content, (B) serum ferritin level. Mice were randomly divided into six groups (blank, B; control, C; 50 mg/kg b.w. DBME, S50; 100 mg/kg b.w. DBME, S100; 200 mg/kg b.w. DBME, S200; desirox group, D) and treated as described in ‘experimental design’ section. Values are expressed as mean ± SD of six mice. *p < 0.05, **p < 0.01, ***p < 0.001 compared with blank and ##p < 0.01, ###p < 0.001 compared with control.

The liver iron content in iron overloaded mice was 133% compared with normal mice. Oral administration of DBME reduced the iron level to 77, 47, and 17% at doses of S50, S100 and S200, respectively (Fig 6A). Ferritin is an intracellular protein responsible for the storage of iron in a non-toxic form. Ferritin also modulates the body’s iron level and helps prevent iron from causing oxidative damage to various cell constituents. Serum ferritin is one of the key markers developed as a consequence of iron overload-induced hepatotoxicity, and the amount of ferritin in the blood indirectly reflects the amount of iron present in the liver. DBME administration notably reduced the serum ferritin concentrations at a similar magnitude to that of the standard (Fig 6B); thus, these data support the iron chelating potency of DBME.

#### Histopathological study

**Fig 7:**
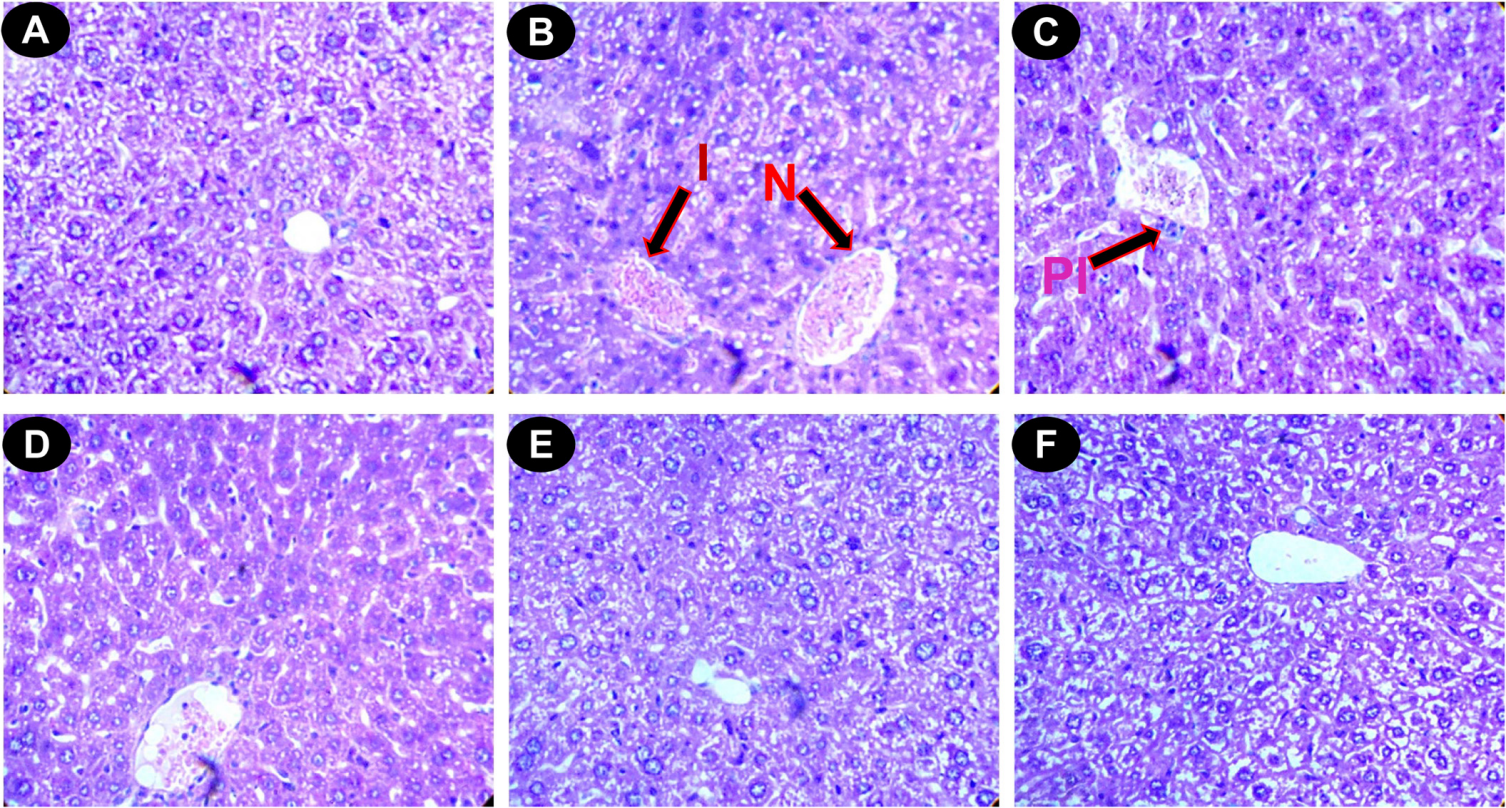
Photomicrograph of mice liver sections (staining with haematoxylin and eosin) ×400. (A) Control mice liver shows normal cellular integrity. (B) Iron-intoxicated (iron dextran, 100 mg/kg b.w.) liver section showing necrosis **(N)**, fatty ballooning degeneration, inflammation **(I)**, and loss of cellular boundaries. (C) Liver section treated with iron dextran + 50 mg/kg b.w. DBME shows improved histology with portal inflammation **(PI)**. (D) Liver section treated with iron dextran + 100 mg/kg b.w. DBME. (E) Liver section treated with iron dextran + 200 mg/kg b.w. DBME. (F) Liver section treated with iron dextran + 20 mg/kg b.w. desirox shows reduced necrotic area and the increased number of hepatocytes. S100 and S200 show reduced hepatocellular necrosis, ballooning degeneration, and inflammation indicating a trend of restoration of normal cellular integrity.

**Fig 8:**
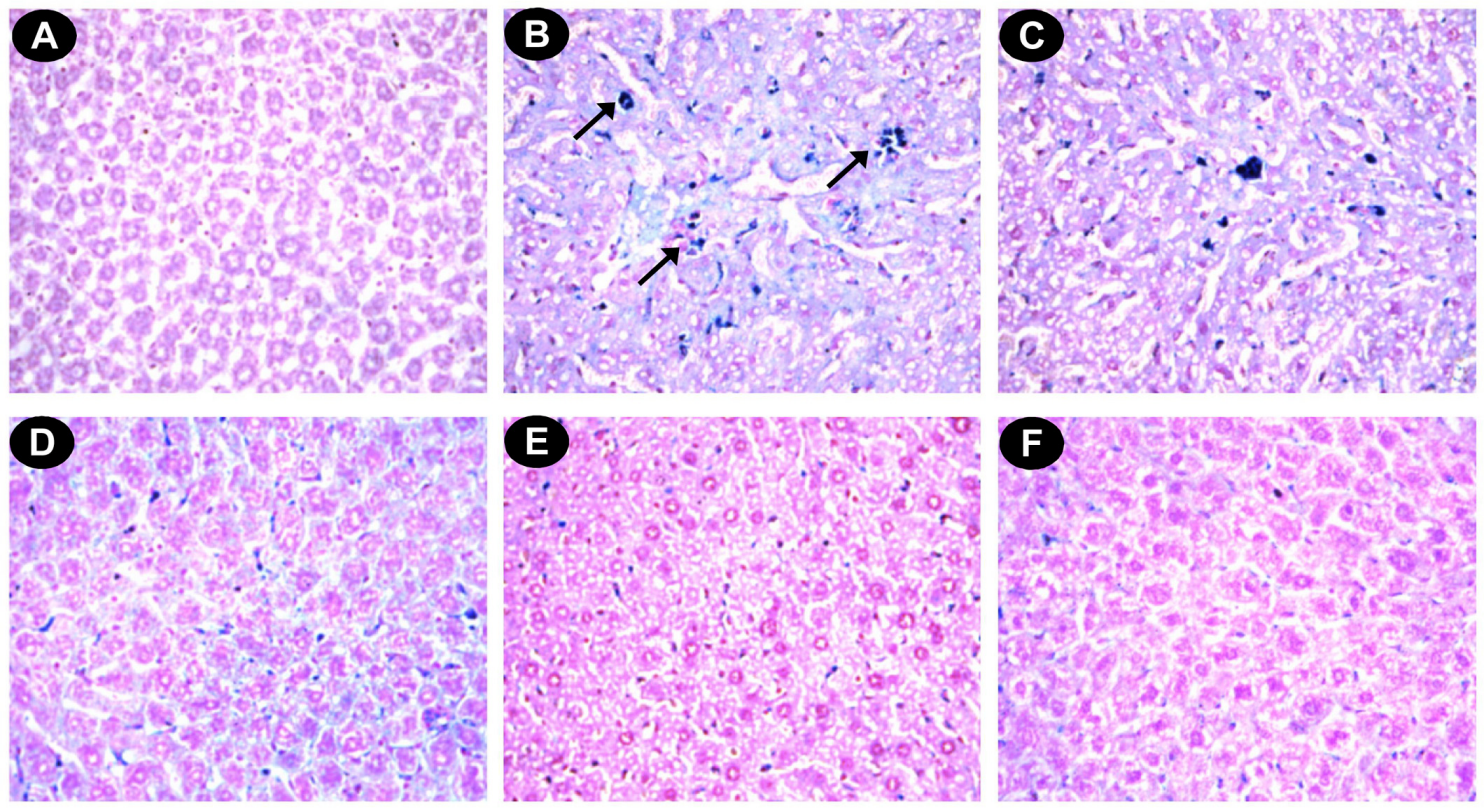
Photomicrograph of mice liver sections (Perls’ staining) ×400. (A) Control mice liver. (B) Iron-overloaded (iron dextran, 100 mg/kg b.w.) liver section showing hemosiderin in blue (indicated by arrows) (C) Liver section treated with iron dextran + 50 mg/kg b.w. DBME shows less hemosiderin. (D) Liver section treated with iron dextran + 100 mg/kg b.w. DBME. (E) Liver section treated with iron dextran + 200 mg/kg b.w. DBME. (F) Liver section treated with iron dextran + 20 mg/kg b.w. desirox shows nearly no hemosiderin. S100 and S200 show very less hemosiderin indicating a trend of restoration of healthy liver with reduced iron content.

**Fig 9:**
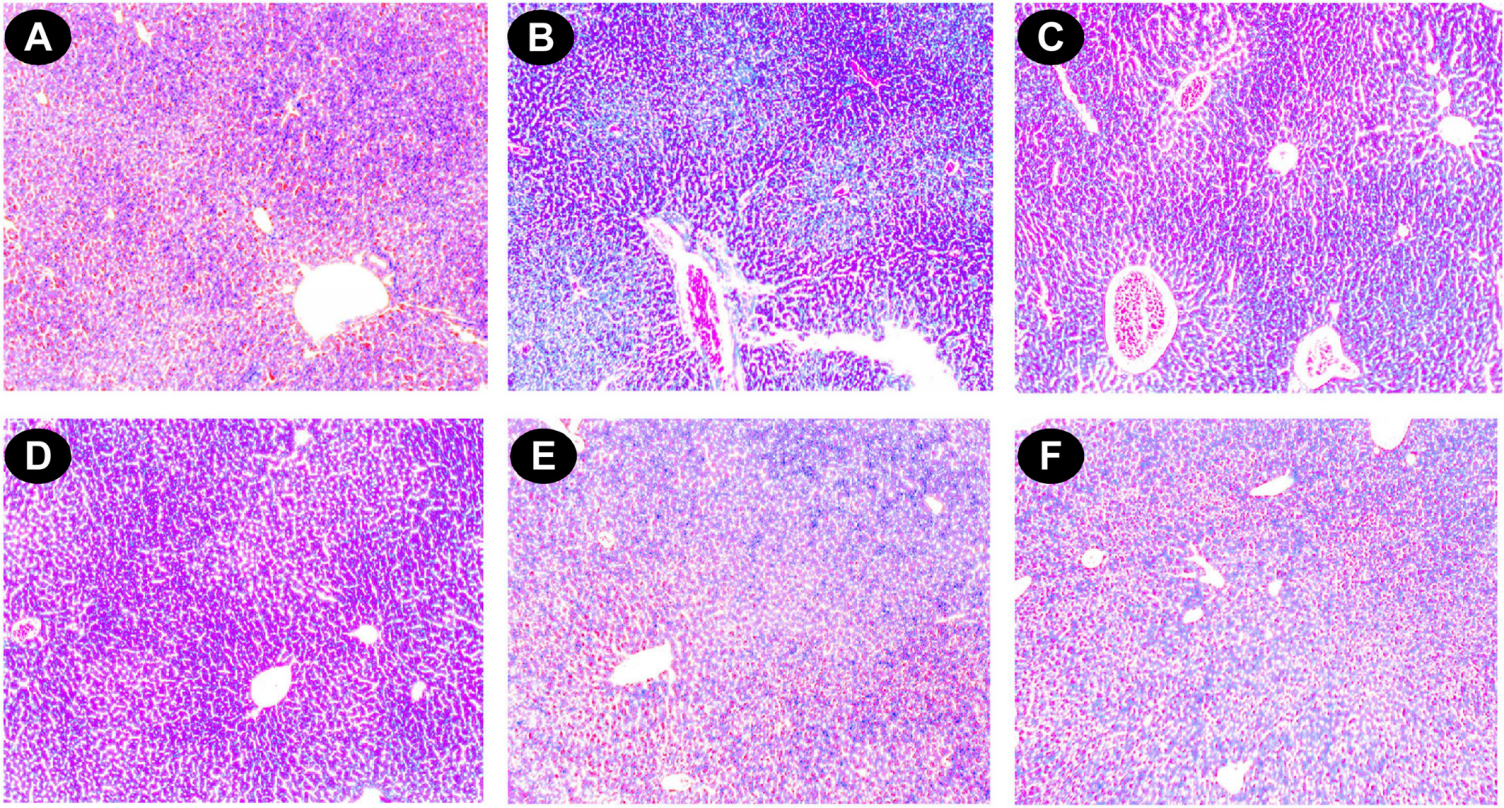
Photomicrograph of mice liver sections (Masson’s Trichrome staining) ×100. (A) Control mice liver shows normal cellular integrity with no fibrosis. (B) Iron-intoxicated (iron dextran, 100 mg/kg b.w.) liver section with elongated fibrous septa and accumulation of collagen (Blue). (C) Liver section treated with iron dextran + 50 mg/kg b.w. DBME shows slight fibrosis. (D) Liver section treated with iron dextran + 100 mg/kg b.w. DBME shows improved histology. (E) Liver section treated with iron dextran + 200 mg/kg b.w. DBME. (F) Liver section treated with iron dextran + 20 mg/kg b.w. desirox shows nearly negligible accumulation of collagen and healthy liver. S100 and S200 show reduced collagen deposition, fibrous septum and necrotic cells in periportal veins indicating a trend of restoration of normal cellular integrity.

The liver sections of normal mice showed regular cell morphology with well-preserved cytoplasm, a prominent nucleus and a well-defined central vein (Fig 7A). Iron overloaded mice showed various pathological changes, including hepatocellular necrosis, ballooning degeneration, inflammation and loss of cellular boundaries (Fig 7B). However, the liver sections taken from DBME-treated mice groups showed evidence of decreased pathogenesis and a marked reduction in hepatic injuries (Fig 7C, 7D and 7E). Fig 7F displays the improved histology of liver sections taken from the desirox-treated group, which is similar to that from the DBME S200 group. The liver sections of the DBME-treated mice showed diffused ballooning degeneration, restored hepatic lesions and reduced neutrophilic cellular inflammation. One of the consequences of iron overload is hemosiderosis, which is marked by the presence of hemosiderin in the liver and other related organs. The iron within hemosiderin deposits is poorly available to the body when needed. Hemosiderin is the complex formed of broken hemoglobin, ferric oxide (unused iron) and ferritin. Perls’ Prussian blue staining is an effective method for the detection of hemosiderin deposition in liver tissue. The liver sections taken from the normal mice showed negligible hemosiderin deposition (blue patches) (Fig 8A). The liver sections taken from the iron overloaded mice showed an increased hemosiderin deposition (Fig 8B). However, the liver sections taken from the DBME-treated mice showed a dose-dependent decrease in the deposition of hemosiderin (Fig 8C, 8D and 8E). The highest dose of DBME (S200) showed similar effects compared with the standard desirox-treated group (Fig 8F). In addition, excessive deposition of iron in liver leads to the liver fibrosis characterized by the proliferation of stellate cells in periportal zones and in association with areas of hepatocellular necrosis. These activated cells shows enhanced procollagen-1 gene expression which results in the increased production of collagen [48]. Masson’s trichrome stain is commonly used to detect fibrotic liver by staining accumulated collagen in liver tissues with blue colour. Results indicate that the liver of control mice shows normal cellular integrity with no fibrosis (Fig 9A). Iron overloaded mice shows elongated fibrous septa and accumulation of collagen (Blue) in periportal zones of liver tissue indicating liver fibrosis state (Fig 9B). However, the liver sections taken from the DBME-treated mice showed a dose-dependent decrease in the collagen accumulation and elongation in fibrous septa (Fig 9C, 9D and 9E). Here also the highest dose of DBME (S200) showed similar instances compared with the standard desirox-treated group (Fig 9F). The results from the histopathological study indicate that DBME-treated mice show a dose-dependent restoration of normal cyto-architecture, suggesting that DBME has potential to ameliorate iron overload induced liver toxicity.

#### Reductive release of ferritin iron and its correlation with reducing power

**Fig 10:**
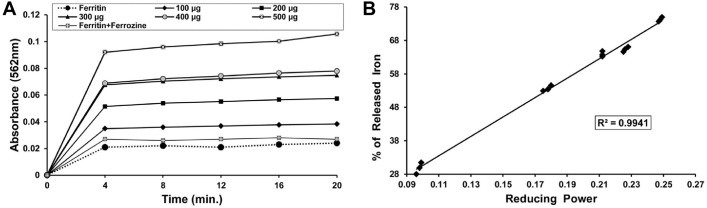
Effect of DBME on reductive release of ferritin iron and its correlation with Fe^2+^reducing power. (A) Reductive release of ferritin iron (B) correlation between released ferritin iron with reducing power. The iron released in response to the increasing amounts (100–500 μg) of DBME was plotted against reducing power displayed by the same doses.Dose dependent formation of the [Fe(ferrozine)_3_]^2+^ complex following release of Fe^2+^ from ferritin by different doses of DBME and single dose of desirox. The reductive release of ferritin iron was quantified by measuring the formation of the ferrous complex of ferrozine, [Fe(ferrozine)_3_]^2+^ at 562 nm using a Shimadzu UV-VIS spectrophotometer.

Various iron chelators are available, and they are administered to attenuate the effects of iron overload. However, these drugs are limited due to the inability to access the iron in ferritin. Therefore, iron chelation therapy is dependent on the reductive release of ferritin iron, which is obtained by addition of a reducing agent, such as ascorbate, to increase the availability of stored iron to chelators [49]. The ability of DBME to reductively release iron from ferritin was quantified by measuring the formation of the ferrous complex ferrozine [Fe(ferrozine)_3_]^2+^. Control experiments without DBME produced negligible amounts of [Fe(ferrozine)_3_]^2+^, whereas DBME dose-dependently increased [Fe(ferrozine)_3_]^2+^ complex formation with time (Fig 10A). In the current study, there is a significant positive correlation (R^2^ = 0.9411) between the reducing power and (%) the iron released from ferritin (Fig 10B). Therefore, DBME can be used as a drug to treat iron overload-induced disease states.

### Identification of probable active compounds in DBME

**Fig 11:**
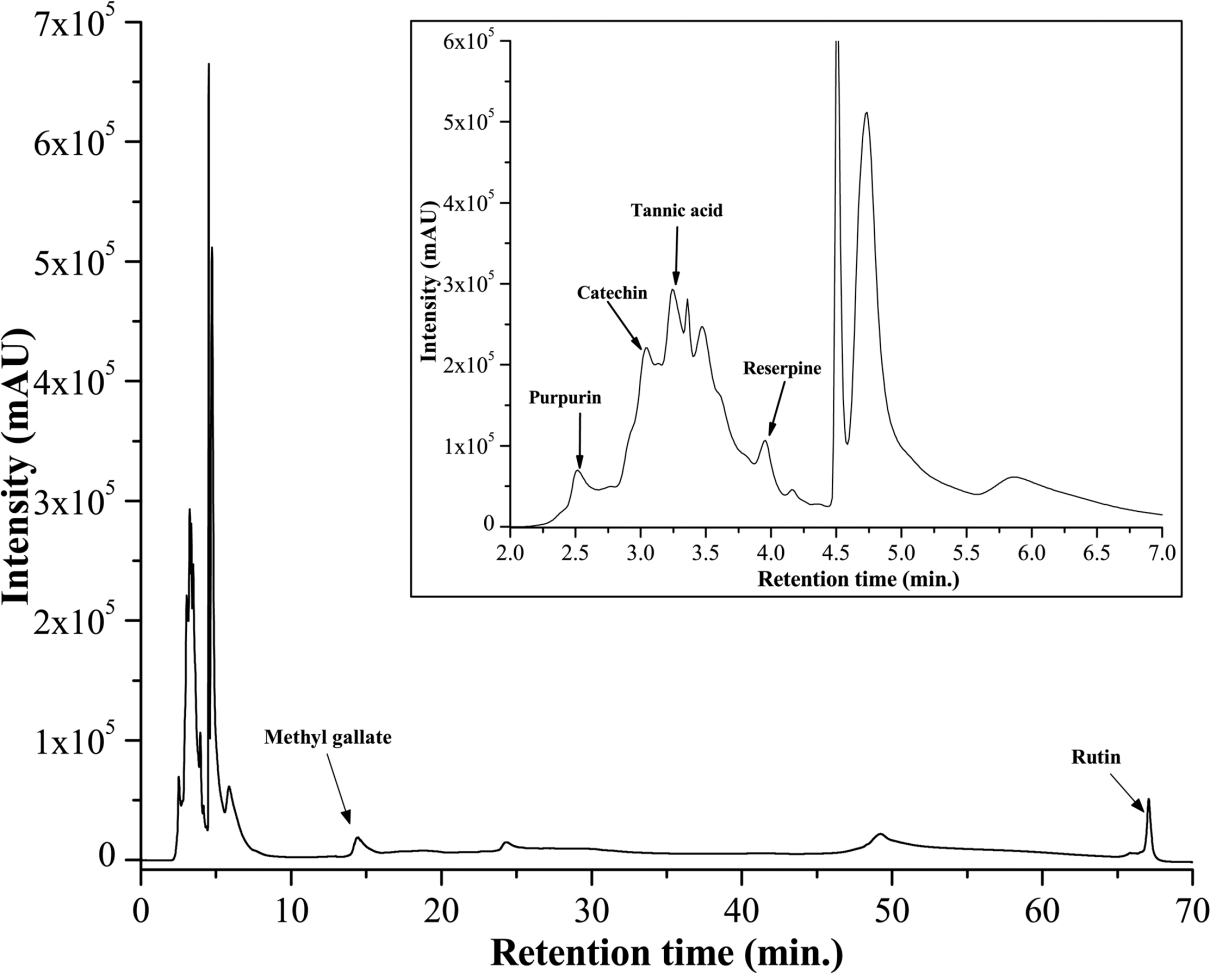
HPLC chromatogram of DBME. Inset shows expanded region of the chromatogram with retention time of 2–6 minutes. Peaks marked signify the retention peak of purpurin (2.5 min), catechin (3.04 min), tannic acid (3.24 min), reserpine (3.97 min), methyl gallate (14.55 min) and rutin (67.08 min).

**Fig 12:**
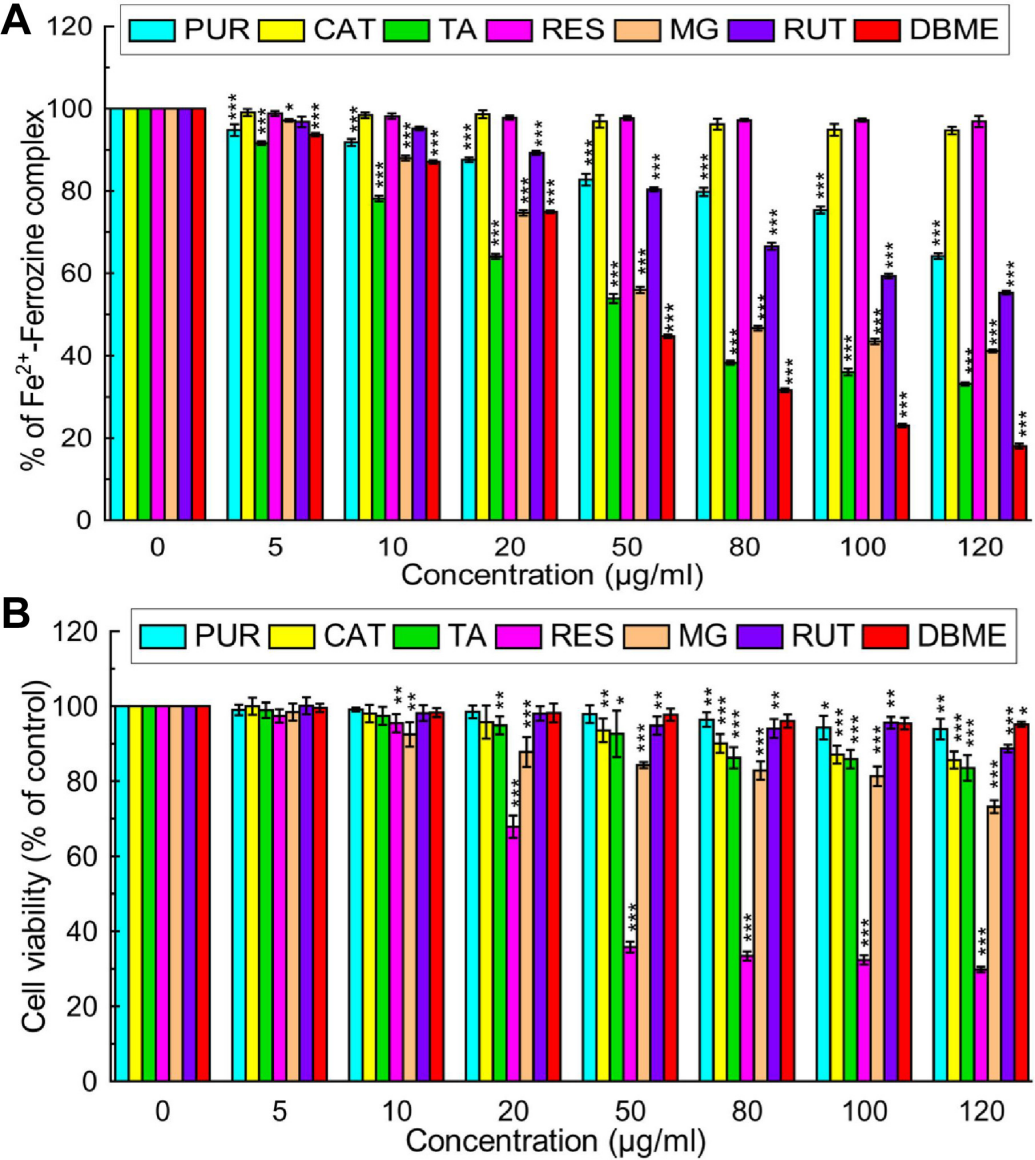
*In vitro* iron chelation activity and cytotoxicity of various compounds and DBME on normal fibroblast (WI-38) cells. (A) *In vitro* iron chelation activity of various compounds. (B) WI-38 cells were treated with increasing concentrations of compounds for 48 hours; cell proliferation and viability was determined with WST-1 cell proliferation reagent. Results were expressed as cell viability (% of control). All data is expressed as mean ± SD (n = 6). *p < 0.05, **p < 0.01 and ***p < 0.001 vs. 0 μg/ml.

To identify the active principle behind the powerful antioxidant and iron chelating activities of DBME, it was subjected to the qualitative and quantitative phytochemical as well as high performance liquid chromatography (HPLC) analysis. The phytochemical analysis showed an abundant amount of phenolics, flavonoids and alkaloids; however, adequate quantities of carbohydrates, tannins and ascorbic acid were also present (Table 4). An HPLC analysis of the sample detected the bioactive compounds in DBME based on the retention time of reference standards. The chromatogram exhibited six main peaks with retention times of 2.5, 3.04, 3.24, 3.97, 14.55 and 67.08 min, which correspond to purpurin, catechin, tannic acid, reserpine, methyl gallate and rutin, respectively (Fig 11). These identified compounds were individually screened for *in vitro* iron chelation ability and cytotoxicity against human normal fibroblast cells (WI-38). Tannic acid was found to be the most effective chelator of Fe^2+^ or inhibitor of the Fe^2+^—Ferrozine complex. The striking IC_50_ value of tannic acid was followed by methyl gallate and rutin. The capacities of these polyphenols to act as antioxidants by chelating or forming a complex with free iron has been established [50]. In addition, purpurin also showed dose-dependent free iron chelating activity. Catechin and reserpine failed to chelate iron at any of the examined concentrations, as corroborated by their IC_50_ values (Fig 12A & Table 5). Based on the cytotoxicity studies, reserpine was the most toxic compound against normal cells, followed by methyl gallate, tannic acid and catechin, which showed differing levels of cytotoxicity in a dose-dependent manner (Fig 12B). Purpurin and rutin showed negligible cytotoxicity against normal cells. By its strikingly good anti-leukemic effect in hybrid mice [51] and its cytotoxic behavior against MCF-7 and A549 [52], reserpine has a history of cytotoxicity against cells of different origins. Speaking of compounds like catechin, tannic acid and purpurin, they are active free radical scavengers [53,54,55]. It is speculated that the probable damage to normal cells caused by compounds such as reserpine is irrespective of its own antioxidant activity [56] is mostly prevented by the array of other antioxidant polyphenols and alkaloids present in the crude extract and at times healed by certain compounds that instigate DNA repair. Tannic acid and methyl gallate show excellent iron chelation activity and a considerable cytotoxicity against normal cells; however, this toxicity was not as potent as the effects of reserpine. On the other hand, DBME displayed a far more promising activity by inhibiting the Fe^2+^—Ferrozine complex formation by more than 80% at its highest dose but found non-toxic to the normal cells at any of examined concentrations. Rutin and purpurin also showed significant inhibition of Fe^2+^—Ferrozine complex formation in a dose-dependent manner and negligible cytotoxicity against normal cells. This varied behavior of individual compounds to chelate free iron and their cytotoxicity against normal cells may be finely tuned in their cocktail mixtures based on their concentrations in the crude extract or synergism with each other or other unidentified phytocomponents. When the components of the crude extract were individually applied, the specificity toward cancer cells was lost and the constituents attacked the normal cells [52], indicating the advantage of using crude extract or mixture of compounds to treat of various diseases. Moreover, it cannot be ignored that the respective percentages in which the compounds co-exist in the plant likely have a vital synergistic role. This mechanism requires further in-depth investigations. Nevertheless, the natural formulation of DBME is a potential safe and effective drug for the treatment of iron overload-induced diseases.

**Table 4:** Quantification of phytochemicals of DBME.

Name of the Assay	DBME
Phenolic content (mg/100 mg extract gallic acid equivalent)	58.69 ± 4.85
Flavonoid content (mg/100 mg extract quercetin equivalent)	61.14 ± 0.16
Carbohydrate content (mg/100 mg extract glucose equivalent)	15.61 ± 0.18
Tannin content (mg/100 mg extract catechin equivalent)	12.18 ± 0.32
Alkaloid content (mg/100 mg extract reserpine equivalent)	95.61 ± 0.81
Ascorbic acid content (mg/100 mg extract L-ascorbic acid equivalent)	3.62 ± 0.05

**Table 5:** IC_50_ values of purpurin, catechin, tannic acid, reserpine, methyl gallate, rutin and DBME for *in vitro* iron chelation and cytotoxicity against WI-38 cells.

Phytochemicals	Iron chelation assay	Cytotoxicity assay
Purpurin	252.37 ± 4.08	1467.75 ± 291.39
Catechin	1940.22 ± 179.61	753.56 ± 31.99
Tannic acid	50.22 ± 0.85	581.03 ± 64.97
Reserpine	3095.13 ± 438.00	44.80 ± 2.06
Methyl gallate	70.86 ± 1.20	344.09 ± 31.34
Rutin	158.96 + 3.86	1230.51 ± 195.42
DBME	40.90 ± 0.31	3351.96 ± 481.04

# All the IC_50_ values are determined in μg/ml. Data expressed as mean ± S.D (n = 6).

## Conclusions

Our *in vitro* studies showed the efficacy of DBME at scavenging different free radicals generated in our body. The alleviation of free iron-induced oxidative stress and hepatotoxicity in mice by DBME was superior to the standard drug desirox. These data confirm the *in vivo* free radical scavenging and hepatoprotective activity of DBME. DBME activity may result from the potential to upregulate antioxidant enzymes and chelate free redox active iron followed by its excretion from the body. The current findings suggest that DBME can be used as an iron chelating drug for the treatment of iron overload-induced diseases, and its activity can be attributed to the bioactive compounds within the extract.

## Supporting Information

S1 Checklist The ARRIVE Checklist. (DOCX)
